# Evolving therapeutic strategies for patients hospitalized with new or worsening heart failure across the spectrum of left ventricular ejection fraction

**DOI:** 10.1002/clc.23849

**Published:** 2022-07-05

**Authors:** John W. Ostrominski, Muthiah Vaduganathan

**Affiliations:** ^1^ Brigham and Women's Hospital Heart and Vascular Center, Harvard Medical School Boston MA USA

**Keywords:** acute heart failure, clinical trials, guideline‐directed medical therapy, heart failure hospitalization, quadruple therapy, registries, worsening heart failure

## Abstract

Heart failure (HF) is a chronic, progressive, and increasingly prevalent syndrome characterized by stepwise declines in health status and residual lifespan. Despite significant advancements in both pharmacologic and nonpharmacologic management approaches for chronic HF, the burden of HF hospitalization—whether attributable to new‐onset (de novo) HF or worsening of established HF—remains high and contributes to excess HF‐related morbidity, mortality, and healthcare expenditures. Owing to a paucity of evidence to guide tailored interventions in this heterogeneous group, management of acute HF events remains largely subject to clinician discretion, relying principally on alleviation of clinical congestion, as‐needed correction of hemodynamic perturbations, and concomitant reversal of underlying trigger(s). Following acute stabilization, the subsequent phase of care primarily involves interventions known to improve long‐term outcomes and rehospitalization risk, including initiation and optimization of disease‐modifying pharmacotherapy, targeted use of adjunctive therapies, and attention to contributing comorbid conditions. However, even with current standards of care many patients experience recurrent HF hospitalization, or after admission incur worsening clinical trajectories. These patterns highlight a persistent unmet need for evidence‐based approaches to inform in‐hospital HF care and call for renewed focus on urgent implementation of interventions capable of ameliorating risk of worsening HF. In this review, we discuss key contemporary and emerging therapeutic strategies for patients hospitalized with de novo or worsening HF.

## 1 INTRODUCTION

Heart failure (HF) is a common, costly, and chronically progressive syndrome characterized by stepwise declines in health status and residual lifespan. Despite significant advancements in both pharmacologic and nonpharmacologic management approaches for chronic HF, acute heart failure (AHF)—whether attributable to new‐onset (de novo) HF or worsening of established HF—accounts for more than one million hospitalizations annually and is a major driver of excess HF‐related morbidity, mortality, and healthcare expenditures.^1^, ^2^, ^3^ Rates of inpatient mortality associated with heart failure hospitalization (HHF) approach 4%–10%, with adverse risk trajectories persisting thereafter.^4^ Approximately one‐fourth and one‐tenth of patients with HHF experience readmission or mortality within 30 days, respectively.^5^, ^6^ The associated patient‐ and societal‐level economic burden of HHF is enormous, accounting for more than half of the cumulative HF‐related expenditures in the United States.^4^ As a result, HHF and worsening HF have emerged as important clinical entities, therapeutic targets, and endpoints in HF clinical trials.^7^, ^8^, ^9^

**Figure 1:**
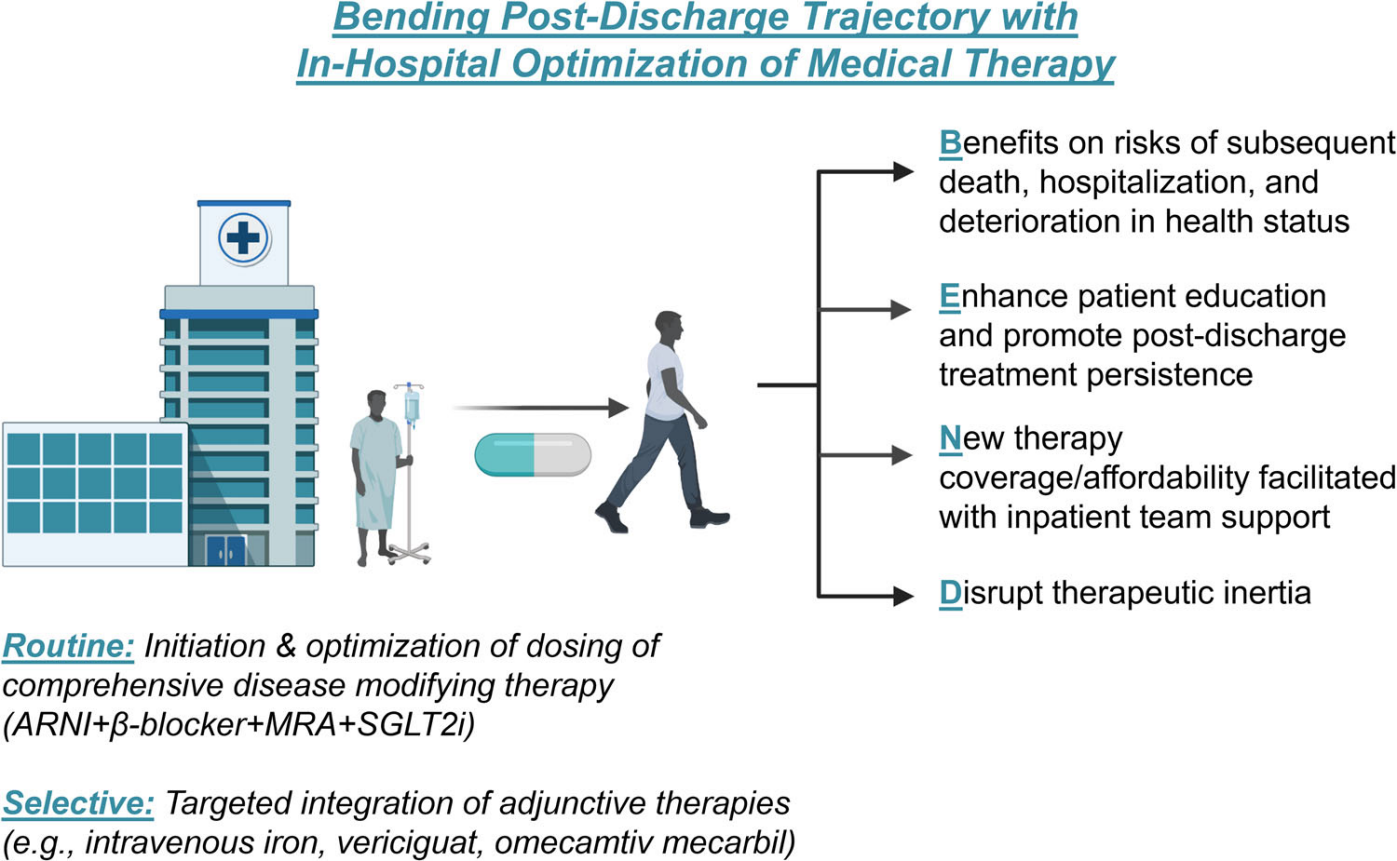
Bending postdischarge trajectory with in‐hospital optimization of medical therapy. Figure constructed in part using BioRender. ARNI, angiotensin receptor neprilysin inhibitor; MRA, amineralocorticoid receptor antagonist; IV, intravenous; SGLT2i, sodium glucose cotransporter‐2 inhibitor.

HHF represents a significant inflection point in the overall trajectory of HF, and therein a potentially high‐yield opportunity to mitigate concurrent and downstream risk. However, even with current standards of care many patients incur worsening in‐hospital clinical trajectories or experience preventable HF rehospitalization in the vulnerable postdischarge period, and these patterns highlight a persistent unmet need for evidence‐based approaches to inform in‐hospital HF care.^6^, ^10^, ^11^ In this review, we discuss key contemporary therapeutic strategies for patients hospitalized with de novo or worsening HF, with a focus on predischarge interventions that improve long‐term outcomes and rehospitalization risk, including initiation and optimization of disease‐modifying pharmacotherapy (Figure 1).

## 2 HOSPITALIZATION FOR DE NOVO HF

### 2.1 Epidemiology and outcomes in de novo HF

Hospitalization for de novo (new onset) HF accounts for approximately 15%–50% of all HHF, and patients with de novo or recently diagnosed HF have distinct clinical risk profiles as compared with patients hospitalized with worsening chronic HF (WHF).^12^, ^13^, ^14^ In a secondary analysis of the ASCEND‐HF trial, patients hospitalized with recently diagnosed HF (≤1 month of admission) were more likely to have younger age, female sex, a nonischemic etiology for HF, HF with preserved ejection fraction (HFpEF), fewer comorbidities, and better post‐discharge survival.^13^ Similar findings have been observed in international registries.^14^, ^15^, ^16^, ^17^ In a study involving over 17 000 patients enrolled in the Danish nationwide registries, de novo HF accounted for 52% of all HHF, and was associated with a 37% lower rate of composite all‐cause mortality or HF readmission when compared with hospitalization for WHF.^14^ Survival outcomes in de novo AHF are similar for HF with reduced ejection fraction (HFrEF) and HFpEF.^16^

Conversely, despite lower rates of major risk factors at baseline, patients admitted to cardiac intensive care units with cardiogenic shock (CS) in the context of de novo HF experience more severe shock and higher rates of in‐hospital mortality as compared with CS in the setting of WHF.^18^ These data highlight the importance of urgent treatment and close attention to in‐hospital trajectory in patients with de novo AHF. The evaluation and management of CS is discussed elsewhere.^19^, ^20^, ^21^

### 2.2 Therapeutic strategies in de novo AHF –time‐sensitive early stabilization

Akin to the time‐sensitive diagnostic and management strategies employed for both ST‐segment myocardial infarction and stroke, the clock for AHF management begins with the initial point of medical contact.^4^, ^22^, ^23^, ^24^ Urgent intervention at this early stage is imperative, as persistent neurohormonal activation, intravascular congestion, hemodynamic perturbations, and systemic inflammation may precipitate preventable myocardial injury and end‐organ dysfunction with implications for near‐ and long‐term death and disability.^3^, ^25^, ^26^, ^27^ However, expeditious diagnosis can be challenging owing to the clinically and hemodynamically protean manifestations of AHF.^22^ Major AHF phenotypes, diagnostic modalities, and common cardiac and noncardiac precipitants are discussed in detail elsewhere.^4^, ^22^, ^24^

As over 80% of patients with AHF are clinically stable and without signs or symptoms of hemodynamic compromise (i.e., “warm and wet”) at presentation,^10^, ^28^ the cornerstone of initial therapy is decongestion. Intravenous loop diuretics are favored owing to their efficacy and rapid onset of action, and urgent therapy is critical. In the REALITY‐AHF registry, a door‐to‐furosemide time of <60 min was associated with 61% lower odds of in‐hospital mortality.^29^ Among patients not receiving loop diuretics at baseline, as is often the case in de novo HF, an initial trial dose of 20–80 mg of intravenous furosemide is typically recommended on the basis of expert opinion.^22^ To ensure attainment of decongestion targets, the diuretic response along with hemodynamic factors and serum electrolytes should be re‐evaluated every 4–8 hours, with adjustments made accordingly. Parallel attention to underlying triggers, complications, and comorbidities should also be given at this stage, as these may either inform or restrict concomitant therapeutic actions. Whenever possible, initial therapies should be directed to the underlying cause of AHF, if apparent.

Adjunctive therapeutic modalities in AHF include supplemental oxygenation, ventilatory support, and vasoactive therapies.^4^, ^22^, ^24^, ^26^ Owing to a paucity of data to guide specific use patterns, these interventions should be tailored to the individual patient and their dominant clinical (e.g., acute pulmonary edema) or hemodynamic (e.g., “cold and wet”) profiles.^4^, ^24^, ^28^ Supplemental oxygen should be reserved for hypoxemic (SpO_2_ < 90% or PaO_2_ < 60 mm Hg) patients as hyperoxia‐induced vasoconstriction may reduce myocardial perfusion.^22^, ^30^ Ventilatory support with either continuous positive airway pressure or noninvasive intermittent positive‐pressure ventilation is associated with more rapid resolution of respiratory distress and metabolic disturbances as compared with standard oxygen therapy among patients with acute cardiogenic pulmonary edema, and improved in‐hospital mortality has been observed in meta‐analysis.^31^, ^32^

Despite extensive prospective investigation of vasoactive therapies in the AHF population, neutral trial results have precluded translation of these modalities into routine practice.^9^, ^26^, ^33^, ^34^, ^35^ Hence, the overall clinical approach has remained largely unchanged over the past several decades, and no therapy has been shown to improve in‐hospital mortality specifically during HHF.^36^, ^37^, ^38^, ^39^ Intravenous vasodilators (i.e., nitrates and nitroprusside) can be considered on the basis of low‐quality evidence to improve symptoms in patients with AHF and preserved systolic blood pressure, but should be used with caution in especially preload‐sensitive states.^22^, ^40^, ^41^ While not recommended as part of usual AHF care due to increased risk of arrhythmia, myocardial ischemia, and mortality,^42^ inodilators (e.g., dobutamine) are key therapies for patients presenting with evidence of hypotension and malperfusion to restore end‐organ perfusion/function, facilitate decongestion, and abrogate shock progression.^21^ Routine use of opioid analgesics is not recommended given the increased risk of mechanical ventilation, prolonged length of stay, mortality, and attenuated antiplatelet effects of oral adenosine receptor antagonists.^43^, ^44^

### 2.3 Therapeutic strategies in de novo AHF —early integration of comprehensive disease modifying therapy

Following initial comprehensive evaluation and implementation of decongestive therapy, ongoing vigilance is needed to ensure the attainment of incremental diagnostic and therapeutic goals. As de novo AHF ultimately represents the end‐pathway of one or multiple disease states, ongoing characterization of the patient and family history, underlying precipitant(s), and myocardial structure/function with as‐needed multimodality imaging may reveal high‐yield therapeutic opportunities and clarify targets for guideline‐directed medical therapy (GDMT). Identification of underlying cardiac (e.g., obstructive epicardial coronary artery disease) or noncardiac (e.g., hemochromatosis) conditions also present specific therapeutic pathways for both HFrEF and HFpEF‐like syndromes.^22^, ^45^ In addition, identification of certain genetic cardiomyopathies, such as hypertrophic cardiomyopathy, may impose unique directives for both indication and timing of cardiac implantable electronic devices (CIED) and familial screening.^46^ Overall, leveraging the index HHF to its full diagnostic and therapeutic potential is imperative to prevent in‐hospital and post‐discharge disease progression, disability, and death.

Although most patients with HHF experience uncomplicated courses, in‐hospital worsening of HF status requiring treatment intensification is observed in 5%–42% of all HHF and is associated with excess mortality and HF readmission.^4^, ^10^, ^11^ As such, the in‐hospital period following initial stabilization constitutes a vulnerable period in the overall course of de novo AHF, warranting timely introduction of disease‐modifying pharmacotherapy to prevent in‐hospital and longitudinal disease progression.^4^, ^47^, ^48^, ^49^, ^50^, ^51^, ^52^, ^53^ Although efficacy and safety data from randomized clinical trials are limited with respect to in‐hospital initiation, sequencing, and titration of GDMT in treatment‐naïve patients with de novo HF, predischarge initiation has largely been shown to be safe, and is associated with substantially improved near‐, intermediate‐, and long‐term outcomes for both HFrEF and HFpEF.^48^, ^51^, ^54^ Hence, for clinically stable patients with resolving AHF, inpatient goals should shift to simultaneous or rapid‐sequence introduction and titration of GDMT tailored to underlying HF phenotype and comorbid conditions, as emphasized by contemporary international clinical guidance documents and performance measures.^4^, ^22^, ^49^, ^50^, ^52^, ^55^, ^56^

In the IMPACT‐HF trial, predischarge initiation of carvedilol in HFrEF was well‐tolerated and associated with a significantly higher rate of treatment at 60 days as compared with post‐discharge initiation.^57^ Differences in favor of carvedilol with respect to mortality and HHF were observed in as early as 14–21 days following treatment initiation in clinically euvolemic patients in the COPERNICUS trial, without differences in adverse events relative to placebo in the first 8 weeks.^58^ Although in‐hospital initiation of bisoprolol or metoprolol succinate has not been prospectively investigated, results in favor of β‐blockers overall have been observed in numerous registry‐based studies.^48^ Collectively, β‐blockers may be safely initiated in‐hospital for clinically euvolemic (i.e., “warm and dry”) patients with HFrEF, with subsequent improvements in long‐term treatment rates and clinical trajectory.^48^, ^50^, ^52^ Low doses are favored initially with gradual titration to evidence‐based doses as tolerated.^49^, ^52^

Inhibitors of the renin–angiotensin‐system, including ACE inhibitors (ACEI), angiotensin receptor blockers (ARB), and angiotensin receptor‐neprilysin inhibitors (ARNI), are foundational therapies for patients with chronic HFrEF.^4^, ^22^, ^59^, ^60^, ^61^ In the Get With The Guidelines‐Heart Failure (GWTG‐HF) registry, in‐hospital initiation of ACEI/ARB was associated with lower 30‐day HF readmission, all‐cause readmission, and mortality, with benefits extending to 12 months post‐discharge.^48^, ^62^ In the PIONEER‐HF trial, in‐hospital initiation of ARNI in stable patients as early as 24  hours after initial presentation with AHF (LVEF ≤ 40%) was safe and associated with substantial and rapid reductions in NT‐proBNP concentration and cardiovascular death or HHF as compared to enalapril.^63^, ^64^ Further, high adherence to ARNI early after discharge is associated with lower rates of readmission and death at 3 and 12 months.^65^ These data indicate in‐hospital initiation of ARNI in patients with stabilized acute HFrEF is both safe and effective even in treatment‐naïve patients without antecedent ACEI/ARB exposure.

In a recent meta‐analysis pooling randomized and observational studies, a modest 9% reduction in all‐cause mortality was observed with ACEI/ARB in HFpEF, without reduction in cardiovascular mortality or HHF risk.^66^ In PARAGON‐HF, sacubitril/valsartan showed a nonsignificant modest benefit on total HHF and cardiovascular death compared with valsartan alone among patients with chronic HF and LVEF ≥ 45%. In this trial, ARNI was well‐tolerated, with discrete benefits among women and patients with LVEF 45%–57%, and patients more recently hospitalized for HF in subgroup and post hoc analyses.^67^, ^68^ Based on PARAGON‐HF, ARNI is approved in chronic HF under the US Food and Drug Administration label^69^ and is being studied among patients who are actively hospitalized in the PARAGLIDE‐HF trial (ClinicalTrials. gov NCT03988634).

Despite clinical practice guidelines supporting the use of mineralocorticoid antagonists (MRA) in chronic HFrEF, significant gaps remain in their use.^22^, ^70^ Although randomized data are presently limited with respect to the overall clinical utility of in‐hospital MRA introduction, observational studies have suggested this practice is safe and supports long‐term adherence, with implications for improved long‐term risk of recurrent HHF and cardiovascular death.^48^ In the ATHENA‐HF trial, there was no difference in 3‐day NT‐proBNP or 30‐day mortality or HHF with in‐hospital use of high‐dose spironolactone (100 mg daily) as compared to usual care alone among patients with stabilized AHF regardless of baseline LVEF.^71^ However, early introduction (within 24 hours of initial intravenous loop diuretic dose) of high‐dose spironolactone was well‐tolerated, including in patients with moderate kidney dysfunction.^71^, ^72^ Taken together, given the established long‐term benefit of MRAs in HFrEF,^22^ CV death and rehospitalization benefits of MRA in HFpEF as observed in TOPCAT‐Americas,^73^ and safety of even high‐dose spironolactone in AHF, hospitalization should be leveraged to advance implementation of this underused therapy.^50^, ^74^

Recently, numerous prospective randomized trials have established the incremental short‐ and long‐term clinical benefit and safety of sodium‐glucose co‐transporter 2 inhibitors (SGLT2i) in both acute and chronic HF. In a meta‐analysis of the DAPA‐HF and EMPEROR‐Reduced trials, SGLT2 inhibition resulted in significant reductions in all‐cause death, cardiovascular death, and HHF among patients with chronic HFrEF.^75^ Early and sustained benefits of SGLT2 inhibition were observed in both trials,^76^, ^77^ with a 58% relative reduction in the risk of death or WHF events only 12 days after initiation.^77^ The EMPEROR‐Preserved trial showed a similarly significant reduction in the combined risk of cardiovascular death or HHF in patients with chronic HFpEF.^78^ Although the precise mechanisms underpinning these observations remain uncertain,^79^ SGLT2i have been shown to confer reverse myocardial remodeling and favorable hemodynamic benefits early after initiation in multiple mechanistic trials,^80^, ^81^, ^82^ as well as rapid and sustained reductions in pulmonary artery pressures among ambulatory patients.^83^ In the EMPA‐RESPONSE‐AHF trial, SGLT2 inhibition was well‐tolerated in stable AHF regardless of LVEF, and significantly reduced the combined risk of WHF, HHF, or death at 60 days.^84^ In the SOLOIST‐WHF trial, although the trial was prematurely terminated due to COVID‐19 related impediments, dual SGLT1/2 inhibition with sotagliflozin significantly reduced risks of total cardiovascular deaths, HHF, and urgent HF visits among patients with diabetes and recent worsening HF. In the EMPULSE trial,^85^ SGLT2i initiation in patients with stabilized AHF was associated with significant clinical benefit and safety at 90 days, including a signal toward reduced all‐cause mortality, HF events, and incidence of acute kidney injury.^86^ Significant improvements in weight and HF‐related symptoms were also noted, and overall benefit was similar regardless of baseline LVEF or de novo/worsening HF status.^86^ Collectively, these data highlight the safety and robust disease‐modifying/reversing potential of SGLT2i in HF across the LVEF spectrum, and have established these agents as key pre‐ and postdischarge therapies for patients with stabilized AHF.^87^ Results from the forthcoming DELIVER (NCT03619213), DICTATE‐AHF (NCT04298229), and DAPA ACT HF‐TIMI 68 (NCT04363697) trials will be expected to further clarify the utility of SGLT2i in HF across LVEF and acuity strata.

Taken together, these data stress the high priority of simultaneous or rapid‐sequence initiation of disease‐modifying therapy during the vulnerable period after initial stabilization for de novo AHF, with favorable downstream implications for treatment adherence, myocardial recovery, health status, recurrent HF events, and ultimately avoidance of preventable deaths.^47^, ^50^ Benefits of each pharmacotherapeutic class appear to be additive, and in many cases one class of therapy (e.g., SGLT2i or ARNI) may enable improved tolerance of agents from another class (e.g., MRA) though preservation of kidney function or maintenance of potassium homeostasis.^49^, ^50^, ^55^, ^88^ In addition, as ARNI, MRA, and SGLT2i have either direct or indirect diuretic effects, tolerance may be improved by initiation during hospitalization.^52^ Safety profiles observed in previously highlighted trials in stable patients are reassuring, and patients may incur incremental mortality risk if crucial pharmacotherapeutic interventions are deferred.^54^ Each agent can be initiated at a low dose with rapid post‐discharge escalation, and the in‐hospital environment leveraged for close monitoring of blood pressure, heart rate, serum electrolytes, and kidney function, keeping in mind that minor fluctuations are expected with many of these agents immediately following initiation and should not automatically prompt discontinuation.

### 2.4 Therapeutic strategies in de novo AHF—inpatient to outpatient transition

Despite the best current systems of care, the early discharge period is characterized by particularly high risk for unplanned recurrent HHF and death.^6^ Hence, the days preceding hospital discharge constitute an important transitional phase and opportunity to address the manifold contributions to residual risk. In particular, this period warrants comprehensive risk reassessment, confirmation of maintenance and rescue diuretic dosing, identification of continuing care clinicians and barriers to adherence, delivery of HF‐focused education to the patient and caregivers, discussion of long‐term goals of care, and development of a plan to address undertreated or untreated comorbidities.^4^, ^6^, ^22^ Although 90‐day and 1‐year rehospitalization rates were lower for de novo HF compared with WHF the IN‐HF Outcome Registry,^89^ patients with de novo HF may be particularly vulnerable to gaps in discharge planning owing to the lack of pre‐existing integration with local systems of care.

Ideally, as patients with de novo HF will often be discharged with multiple new medications, repeat monitoring of kidney function and serum electrolytes should be performed in 7 days post‐discharge or earlier in event of instability predischarge. The initial outpatient follow‐up should occur no later than 2 weeks following discharge, ideally earlier in patients with de novo HF reassess volume status, reinforce education, and titrate GDMT. Although the extent of adverse remodeling at presentation may have implications for myocardial recovery, a repeat assessment of left ventricular systolic function should occur no earlier than 90 days following optimization of medical therapy in patients with de novo HFrEF to guide routine decisions regarding primary prevention cardioverter defibrillator implantation, as significant reverse remodeling in the setting of optimal medical therapy may preclude this need.^46^, ^50^, ^59^, ^81^, ^90^

Although patients with de novo HF generally exhibit better cardiorespiratory fitness when compared to those with WHF,^91^ health status at discharge in AHF is highly predictive of near‐ and long‐term risk of recurrent HHF and cardiovascular death.^92^ Hence, all patients should be referred to an HF‐focused cardiac rehabilitation (CR) program at the time of discharge.^22^ Participation in CR is associated with substantial improvements in physical function, quality of life, HHF risk, and all‐cause mortality among HF patients regardless of LVEF, age, frailty, or comorbidity burden.^93^, ^94^, ^95^, ^96^ Despite this, CR remains highly underutilized; in the GWTG‐HF registry, only one‐tenth of eligible HF patients received CR referral after HHF.^97^, ^98^, ^99^

Multimorbidity is prevalent in the HHF population, is increasing with time, and contributes adversely overall health status, recurrent HHF, and mortality risk.^22^, ^100^, ^101^ Hence, a comprehensive strategy should be developed to address treatment of overt, latent, and foreseeable cardiac and noncardiac comorbidities before discharge. The overall lower comorbidity burden observed in de novo HF highlights an important opportunity for primary and secondary prevention efforts, as incident comorbid conditions may not only contribute independently to HF progression but also influence tolerance of established therapies (e.g., incident chronic kidney disease [CKD]) and candidacy for advanced therapies. Further, comprehensive treatment protocols should encourage all appropriate vaccinations, as these are preventable systemic insults capable of provoking cardiac decompensation with resultant morbidity and mortality.^22^

Finally, despite under‐inclusion in extant clinical guidance documents,^102^ considerations of cost and value are increasingly important to consider at the bedside in an era of increasing HF‐related societal costs and patient‐level financial burden.^103^ Financial toxicity occurs in a substantial proportion of patients with HF, and is associated with detrimental health impact.^104^, ^105^ Patients with HF are generally receptive to cost‐based conversations,^106^ and discharge prescriptions should account for expected out‐of‐pocket costs after exhausting all possible opportunities for cost‐mitigation.^52^ Emerging pragmatic techniques, including a recently proposed spending function,^107^ are potential conceptual frameworks to consider the patient‐specific value of a new therapy.

## 3 HOSPITALIZATION FOR WORSENING HF

### 3.1 Epidemiology and outcomes in WHF

Hospitalization for WHF—defined as worsening HF signs and symptoms in a patient with chronic HF requiring intensification of therapy after a period of clinical stability—comprises the majority of all HHF in most settings, and contributes to accelerated disease progression, excess mortality, and enormous financial burden.^7^ Overall, in‐hospital and post‐discharge outcomes for WHF are worse as compared with de novo HF for both HFpEF and HFrEF, likely owing to a higher‐risk phenotype characterized by older age, lower baseline health status, higher medical complexity, and undertreatment. In the IN‐HF Outcome Registry, 1‐year all‐cause mortality, cardiovascular mortality, and HF‐related rehospitalization were nearly two‐ to threefold higher for WHF as compared with de novo HF, regardless of LVEF.^89^ In the NCDR PINNACLE registry, rates of 30‐day readmission and 2‐year mortality after hospitalization with worsening HFrEF were 56% and 23%, respectively, with risk especially concentrated in older and multimorbid patients.^108^ At the onset of WHF, 42% of patients were receiving monotherapy, 43% receiving dual therapy, and only 14% receiving triple therapy, highlighting a risk‐treatment paradox that has been observed in other registries and in other forms of established cardiovascular disease.^69^, ^107^, ^108^, ^109^

### 3.2 Therapeutic strategies in WHF—time‐sensitive early stabilization

The overall approach to initial stabilization in WHF is similar to that previously described for de novo HF—relying primarily on prompt relief of congestion coupled with as‐needed correction of hemodynamic perturbations and treatment of identifiable precipitants—with key differences relating to initial decongestive therapy and management of baseline GDMT. In patients receiving loop diuretic therapy at baseline, a proposed initial dosing regimen could be 2.5 times the baseline total daily dose in furosemide equivalents, an approach shown to be safe and effective in the DOSE trial.^4^, ^111^, ^112^ Approximately 20% of patients require escalation or re‐initiation of decongestive therapies, potentially driven by either under‐dosing of initial therapy or diuretic resistance.^10^, ^111^ To overcome these, escalation of loop diuretic therapy may be warranted for patients with persistent congestion and may be tailored to goal total urine output (≥150 cc/h) or an emerging strategy of spot urine sodium excretion (≥50–70 mEq/L).^111^ Additional measures include supine positioning,^113^ combination therapy,^114^, ^115^ vasodilatory and inotropic therapies,^11^, ^116^ and ultrafiltration.^22^

Approximately 10%–15% of patients present with signs and symptoms of acute HF/CS requiring immediate consideration of vasoactive agents, invasive assessment of hemodynamics, or advanced therapies including mechanical circulatory support.^4^, ^10^ Increased vigilance for identification and treatment of this subset is often needed in the WHF population, as higher rates of undertreatment and baseline end‐organ dysfunction generally portend impaired physiologic reserve and lower threshold for onset and perpetuation of the shock spiral. Additional clues to these especially high‐risk groups are highlighted elsewhere.^4^, ^22^

Baseline GDMT should generally be continued at admission for WHF in the absence of high‐risk bradyarrhythmia, hemodynamic instability, or acute kidney injury. Residual concern may exist regarding the negative inotropic effects of β‐adrenergic antagonism in AHF, but data from prospective trials and observational studies have shown that continuation of β‐blockers is associated with improved postdischarge adherence and clinical outcomes.^117^, ^118^, ^119^ Although SGLT2i have been shown to be safe when introduced during HHF, severe acute illness, planned near‐term surgical intervention, or anticipated prolonged fasting may prompt temporary holding in high‐risk patients to limit risks of diabetes ketoacidosis.^119^, ^120^, ^121^

### 3.3 Therapeutic strategies in WHF—therapeutic optimization with comprehensive disease modifying therapy

Regimented reassessment of clinical trajectory is particularly important in patients admitted with WHF owing to their higher‐risk profile, and identification of risk‐enhancing trajectories should prompt escalation of current therapy, consideration of advanced diagnostics (e.g., invasive hemodynamic assessment) and therapeutics, and re‐consideration of overall goals of care.^4^ This phase of care may also reveal high‐yield opportunities for diagnostic and therapeutic optimization, including performing key diagnostic procedures that may have been previously omitted or deferred but have important relevance for subsequent survival. For instance, although myocardial revascularization among patients with ischemic cardiomyopathy is associated with reduced disease progression and mortality risk,^22^, ^123^ substantially fewer than half of all patients with de novo HFrEF presenting across Veterans Affairs Healthcare System hospitals underwent an ischemic evaluation, with considerable variation between centers.^124^ Hence, a sizeable proportion of patients with recurrent HHF may present with important diagnostic opportunities.

Given the established benefits and underuse of GDMT among patients with WHF, every opportunity should be leveraged for therapeutic optimization before discharge. In a real‐world cohort, treatment intensification during HHF was associated with substantially improved survival and rehospitalization risk at 12 months, while post‐discharge treatment intensification was uncommon.^125^ Among patients with stabilized worsening HFrEF, evidence‐based β‐blockers and MRAs should be initiated and/or escalated to target doses. For those either receiving therapy with ACEI/ARB at baseline or naïve to RAAS inhibitors, switching to or initiation of ARNI, respectively, is preferred owing to improved clinical outcomes regardless of whether recently hospitalized.^22^, ^60^, ^63^, ^64^, ^126^ A 36‐h washout period is required before ARNI initiation in patients previously on ACEI to mitigate risks of angioedema. Prospective trials evaluating SGLT2i in concurrent WHF, recent WHF, and chronic HF have revealed significant and early clinical benefit across the LVEF spectrum, with greater absolute risk reductions observed among recently hospitalized patients.^50^, ^76^, ^78^, ^86^, ^127^ These observations support consideration of SGLT2i initiation in all patients with WHF.

Several practical considerations are worth noting for deployment of these lifesaving therapies in clinical practice. Although hypotension remains source of clinical hesitancy, MRA and SGLT2i have had virtually no important impact on systolic blood pressure (SBP) in clinical trials among those with lower baseline BP. Although ARNI introduction is associated with more significant hypotension and orthostasis, it is usually tolerable and safe with appropriate patient education. Evidence‐based β‐blockers without α adrenergic effects may also be prioritized to augment tolerance of other agents. The risk of incident hyperkalemia may be managed by transitioning from ACEI/ARB to ARNI and initiation of SGLT2i, which both have been shown to reduce hyperkalemia risks. Introduction of potassium binders may further augment tolerance of RAAS inhibitors,^22^, ^128^ a strategy being investigated in the REALIZE‐K (NCT04676646) and LIFT (NCT05004363) trials, and recently shown to be effective in DIAMOND (NCT03888066). CKD constitutes an important comorbidity for those facing worsening HF; however, evidence and labeling have supported use of therapies such as the SGLT2i down to eGFR 20 ml/minute/1.73 m^2^.^122^ The safety and efficacy of SGLT2i in hemodialysis populations is being in the RENAL LIFECYCLE and EMPA‐HD (NCT05179668) trials.

### 3.4 Therapeutic strategies in WHF—tailored management approaches

Importantly, variation in comorbidity composition, tolerance barriers, and residual risk despite maximal optimization may mandate consideration of adjunctive therapeutic options.^61^ Intravenous iron may be considered in symptomatic patients with HF, LVEF ≤ 50%, and recent or concurrent hospitalization meeting trial criteria for iron deficiency (serum ferritin < 100 ng/ml or 100–299 ng/ml with TSAT < 20%) to improve health status and rehospitalization risk.^22^, ^129^, ^130^ Of note, patients with TSAT < 20% and serum iron < 13 μmol/L appear to have a particularly high risk of 5‐year mortality.^131^ Digoxin is also a viable adjunctive therapy capable of improving hemodynamic status and all‐cause rehospitalization risk in patients with HFrEF, and may be especially favored among patients with comorbid atrial fibrillation (AF) provided close monitoring for toxicity can be reliably performed.^6^, ^49^ In patients with an LVEF ≤ 35% in sinus rhythm who have a resting heart rate ≥70 beats per minute despite a maximally tolerated β‐blocker or intolerance/contraindication to β‐blocker therapy, ivabradine (I_*f*_ channel inhibitor) may be considered to reduce HHF burden and HF‐related death.^22^, ^132^ Fixed‐dose hydralazine and isosorbide dinitrate may be considered among Black adults with symptomatic HFrEF who are already optimized on other elements of guideline‐directed medical therapy.^4^, ^22^

Vericiguat (soluble guanylate cyclase stimulator) and omecamtiv mecarbil (cardiac myosin activator) have also demonstrated modest relative but greater absolute benefits in reducing readmission risks among high‐risk patients with worsening HFrEF, although inclusion of the latter in the current generation of HF‐focused guidelines is limited by lack of regulatory approval.^22^, ^61^, ^133^, ^134^ Once both are regionally approved and accessible, these therapies will be important adjuncts for implementation in this high‐risk subset of worsening HF. Omecamtiv mecarbil may have uniquely favorable properties in this setting given that it is relatively hemodynamically neutral and may especially match a very high‐risk patient profile (who often face intolerance to other core therapies).

In addition to adjunctive pharmacotherapeutic interventions, several forms of procedural and device‐based intervention have discrete and emerging utility for reduction of HF‐related morbidity, mortality, and rehospitalization risk, and candidacy should be considered for all patients with WHF. AF ablation should be considered in patients with concomitant HFrEF and AF owing to significant reduction in the combined risk of clinical outcomes in the CASTLE‐AF trial.^135^ Cardiac resynchronization therapy should be performed for patients with HFrEF (LVEF ≤ 35% despite optimal medical therapy) and chronic left bundle branch block (QRS > 150ms) or LVEF < 50% with an indication for permanent ventricular pacing.^22^, ^136^ His bundle pacing may be a future modality in this group.^137^ For appropriately selected patients with HFrEF, refractory symptoms despite maximally titrated GDMT, and significant functional mitral (and potentially tricuspid) regurgitation, transcatheter edge‐to‐edge repair (TEER) should be considered in accordance with extant HF and valvular heart disease guidelines.^22^, ^138^, ^139^, ^140^ Further, although the recent REDUCE LAP‐HF II trial did not show the benefit of an interatrial shunt device among patients with symptomatic HF with LVEF ≥ 40%, prespecified subgroup analysis disclosed benefit among patients with a peak exercise pulmonary vascular resistance of <1.74 Wood units, potentially representing a responder group meriting dedicated prospective evaluation.^140^ Finally, invasive remote monitoring techniques, such as implantable wireless pulmonary arterial pressure sensors (e.g., CardioMEMS), CIED‐based hemodynamic monitors, and novel wearable biosensors can provide near‐real‐time diagnostic information enabling long‐term avoidance of HF rehospitalization and associated morbidity.^142^, ^143^ Wireless pulmonary arterial pressure sensors offer potential protection against rehospitalization among symptomatic patients with HFrEF, but optimal patient selection and cost‐effectiveness remain unclear.^144^

### 3.5 Therapeutic strategies in WHF—inpatient to outpatient transition

As compared to de novo HF, the early postdischarge period following hospitalization for WHF is characterized by extreme vulnerability owing to worse short‐ and long‐term outcomes, with rates of all‐cause rehospitalization and mortality exceeding 60% by 1 year and 85% by 5 years.^145^ Further, in the PIONEER‐HF trial, more than half of all patients required escalation of diuretic therapy within 6 weeks of discharge.^63^ Akin to the transitional strategy discussed for de novo HF, important goals for WHF include facilitating focused discharge handoffs to continuing care clinicians (including noncardiovascular subspecialists), HF re‐education, and ongoing treatment of undertreated or untreated comorbidities. Close outpatient monitoring and follow‐up to verify tolerance are particularly important, as higher rates of comorbid conditions and polypharmacy may confound adherence due to limited hemodynamic, electrolyte, and financial reserves.^107^ Acknowledging the poor outcomes post‐WHF, particularly among patients with advanced age,^108^ greater frailty,^4^ socioeconomic deprivation,^146^ persistent symptoms at discharge,^92^ or in patients for whom GDMT was de‐escalated during hospitalization,^48^, ^147^ comprehensive risk assessment should include conversations about advance care planning and goals of care, with consideration for referral to advanced HF and palliative care subspecialists.

## 4 CONCLUSION

Despite significant advancements in both pharmacologic and nonpharmacologic management approaches for chronic HF, more than one million HF hospitalizations occur annually in the United States, each contributing to excess HF‐related morbidity, mortality, and healthcare expenditures. Hospitalizations for de novo and worsening HF are distinct clinical entities with unique therapeutic and prognostic considerations. Available data support hospitalization as a high‐yield opportunity to favorably influence the overall HF trajectory through rapid‐sequence initiation of disease‐modifying pharmacotherapies before discharge. High‐risk patients facing worsening HF may be further optimized with targeted drug and device therapies, including intravenous iron, vericiguat, and omecamtiv mecarbil. Key goals of hospitalization should move beyond attention to decongestion and focus on averting downstream disease progression, rehospitalization, and their contribution to preventable death and disability.

## CONFLICTS OF INTEREST

Muthiah Vaduganathan has received research grant support or served on advisory boards for American Regent, Amgen, AstraZeneca, Bayer AG, Baxter Healthcare, Boehringer Ingelheim, Cytokinetics, Lexicon Pharmaceuticals, Novartis, Pharmacosmos, Relypsa, Roche Diagnostics, and Sanofi, speaker engagements with Novartis and Roche Diagnostics, and participates on clinical trial committees for studies sponsored by Bayer AG, Galmed, Occlutech, Novartis, and Impulse Dynamics. The remaining authors declare no conflict of interest.

## Data Availability

No primary previously unpublished data are presented in this review.
